# Supplementary material for: Genome sequencing sheds light on the contribution of structural variants to Brassica oleracea diversification
Source: BMC Biol. 2021 May 5;19:93. doi: 10.1186/s12915-021-01031-2 (PMC8097969; doi:10.1186/s12915-021-01031-2)
Supplement: Supplementary file 1 — Additional file 1: Figure S1. Morphology of cauliflower Koroso_1401 and pointed cabbage OX-heart_923. Figure S2. Genome size estimation using flow cytometry analysis for cauliflower Korso and cabbage OX-heart. Figure S3. Chromosome conformation capture of cauliflower Korso and pointed cabbage OX-heart. Figure S4. Pairwise comparisons of the pseudo-chromosomes among cauliflower Korso, cabbage OX-heart and broccoli HDEM. Figure S5. Distribution of estimated insertion times of intact LTR retrotransposons in Korso, OX-heart and B. rapa (v3.0) genomes. Figure S6. Distribution of centromeric satellite repeats CentBr 1 and CentBr 2 on the nine chromosomes of Korso and OX-heart. Figure S7. Segmental collinearity between the A. thaliana genome and genomes of Korso, OX-heart and B. rapa. Figure S8. Subgenomes in Korso and OX-heart. Figure S9. Detected SVs between the genomes of Korso and the OX-heart. Figure S10. Significantly enriched GO terms in genes overlapping with detected SVs in their gene body and/or promoter regions. Figure S11. Distribution of SV genotyping rates in 271 B. oleracea accessions. Figure S12. Population analysis of the 271 B. oleracea accessions using SNPs. Figure S13. Manhattan plot displaying the -log(q-value) of allele variations between cabbage and cauliflower for SVs across the Korso genome. Figure S14. Expression profiles of genes potentially involved in curd maintenance and floral arrest. [file 12915_2021_1031_MOESM1_ESM.pdf]

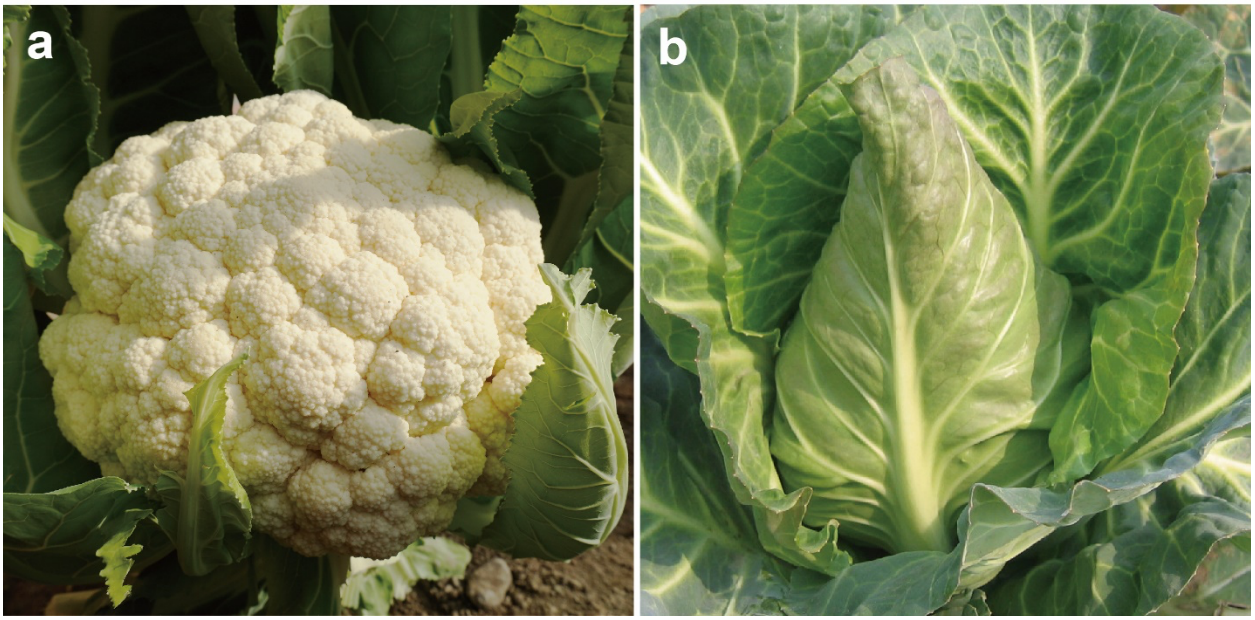

Figure S1 Morphology of cauliflower Koroso_1401 (**a**) and pointed cabbage OX-heart_923 (**b**).

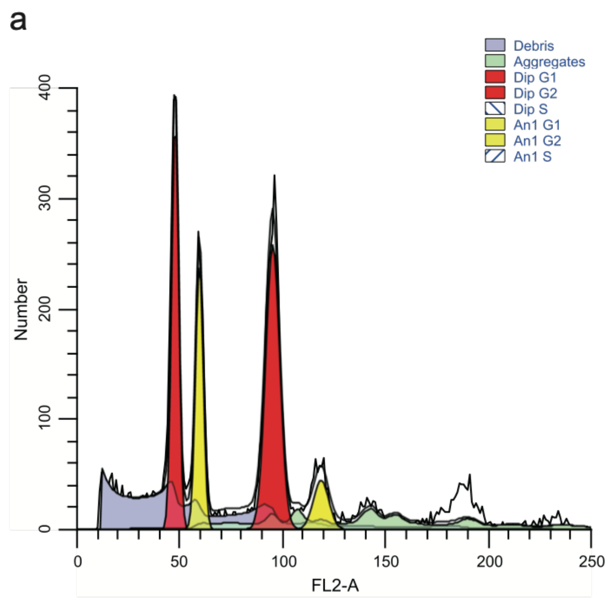

Figure S2 Genome size estimation using flow cytometry analysis for cauliflower Korso (**a**) and cabbage OX-heart (**b**). *B. rapa* Chiifu-401-42 was used as the reference. Red peaks represent the reference *B. rapa*, and yellow peaks represent Korso and OX-heart in (**a**) and (**b**), respectively. Genome sizes of Korso and OX-heart were calculated according to the relative positions of the peaks with the reference *B. rapa*.

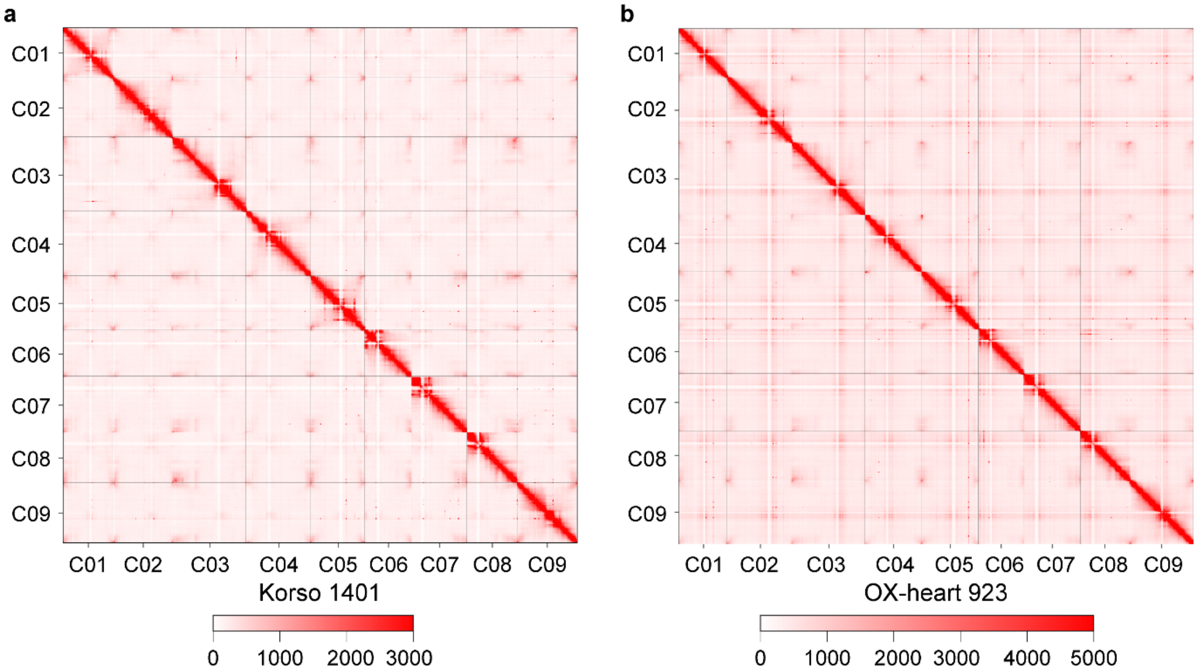

Figure S3 Chromosome conformation capture of cauliflower Korso and pointed cabbage OX-heart. **a**,**b**, Hi-C interaction heatmaps of Korso (**a**) and OX-heart (**b**) genomes. The colored bars indicate the intensity of interaction, which represents the normalized count of Hi-C links between 1-Mb bins.

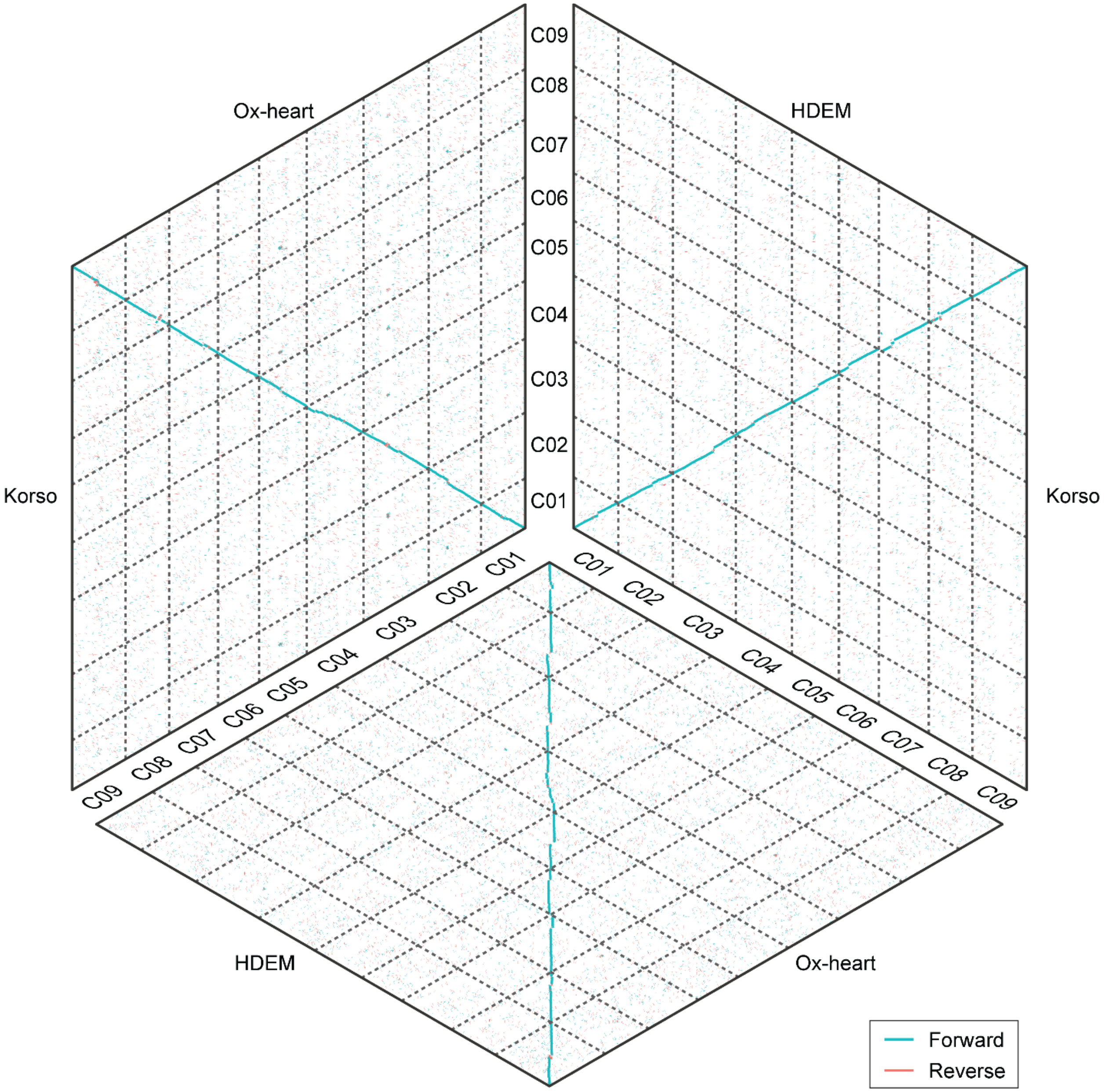

Figure S4 Pairwise comparisons of the pseudo-chromosomes among cauliflower Korso, cabbage OX-heart and broccoli HDEM.

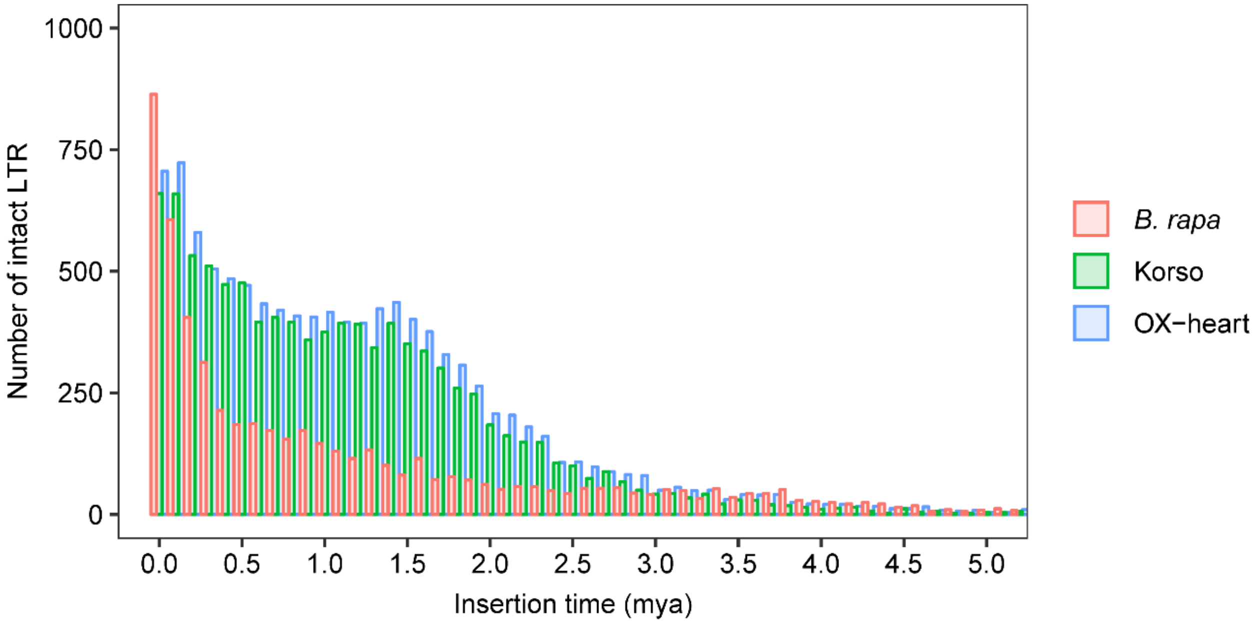

Figure S5 Distribution of estimated insertion times of intact LTR retrotransposons in Korso, OX-heart and *B. rapa* (v3.0) genomes. MYA, million years ago.

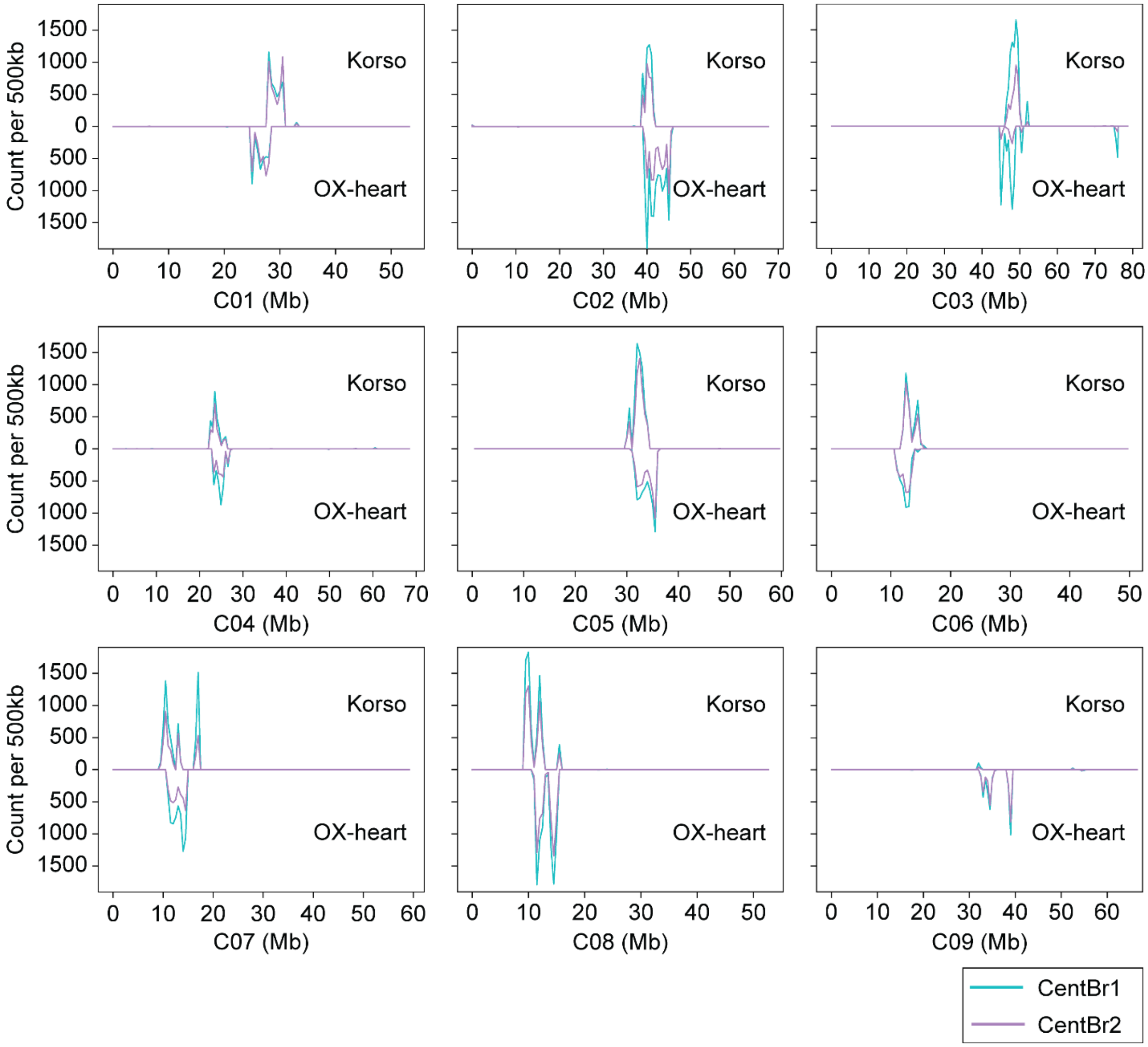

Figure S6 Distribution of centromeric satellite repeats CentBr 1 and CentBr 2 on the nine chromosomes of Korso and OX-heart.

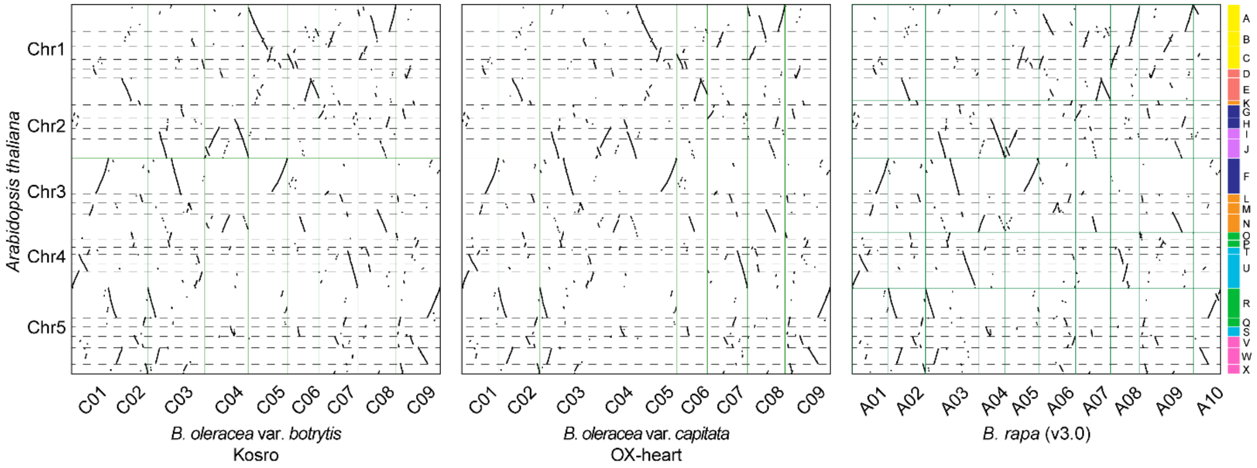

Figure S7 Segmental collinearity between the *A. thaliana* genome and genomes of Korso, OX-heart and *B. rapa*. The conserved collinear blocks labeled A to X are color-coded by inferred ancestral chromosomes according to Wang et al. (2011).

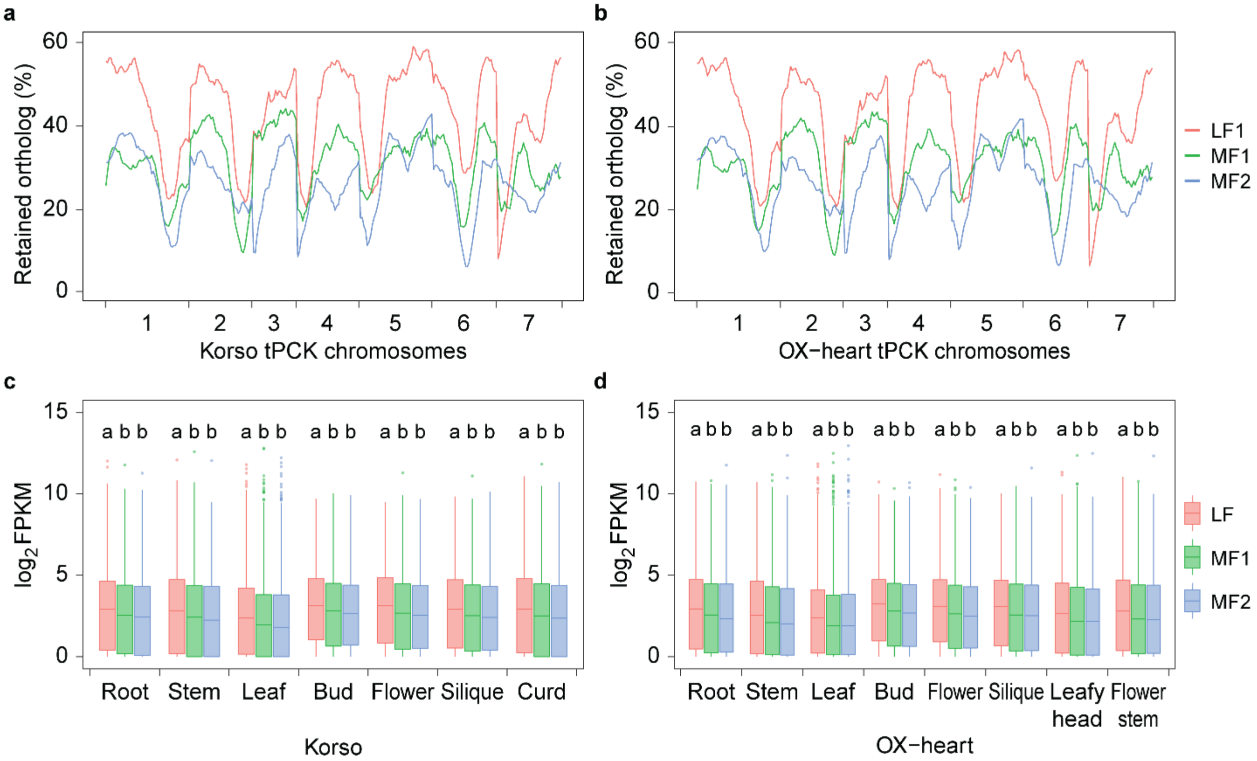

Figure S8 Subgenomes in Korso and OX-heart. **a**,**b**, Percentage of retained gene orthologs in the three subgenomes (LF, MF1 and MF2) of Korso (**a**) and OX-heart (**b**). The x axis denotes the seven supposed translocated Proto-Calepine Karyotypes (tPCK1-7). **c**,**d**, Expression of genes with orthologs identified in all three subgenomes in different tissues of Korso (**c**) and OX-heart (**d**). Group labeled with different letters indicate significant difference at P < 0.05 (Tukey's HSD test).

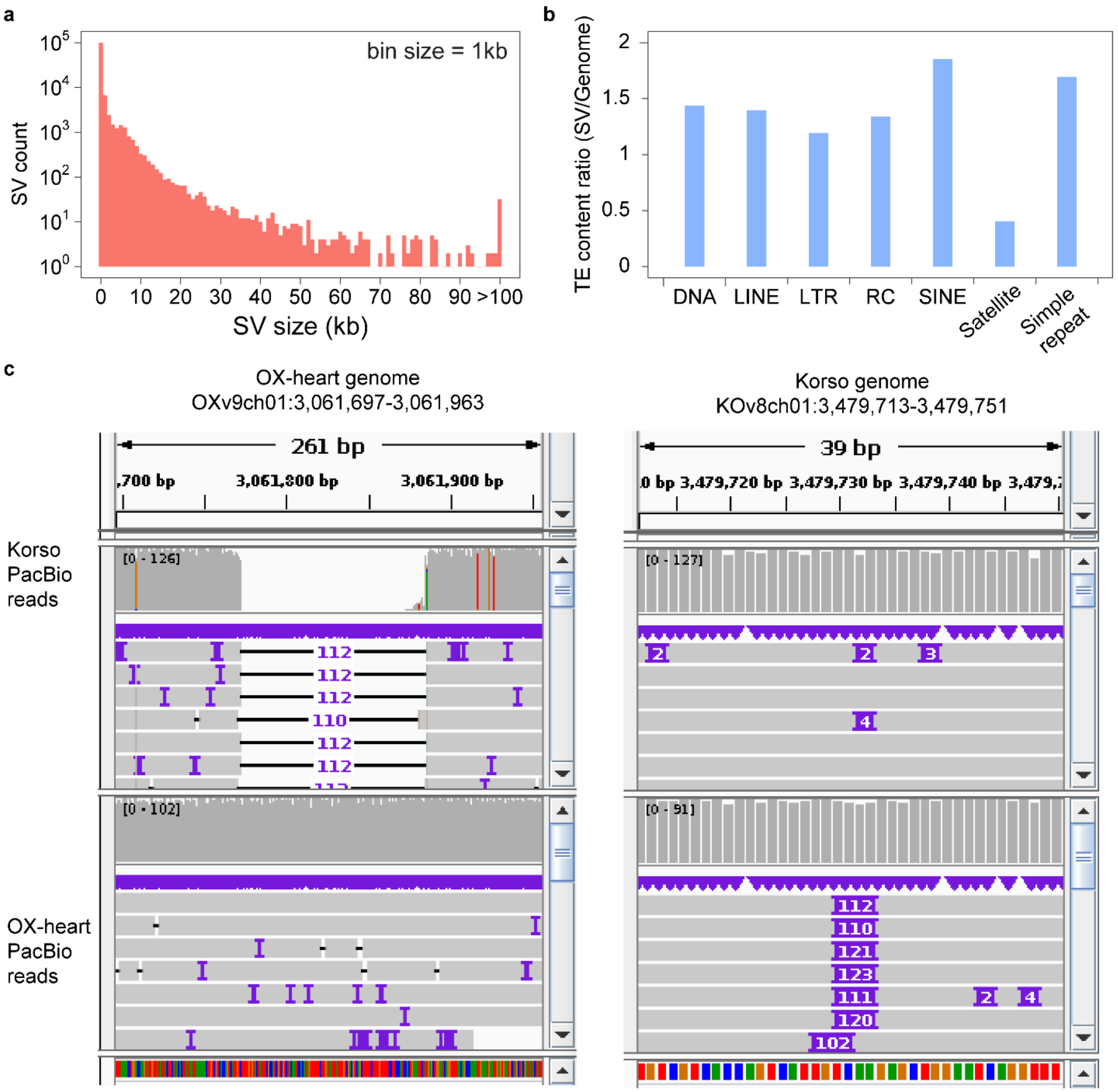

SV		Ox-heart genome				Korso genome			
ID	Type	Chr	Start	Stop	Size	Chr	Start	Stop	Size
SV_w_3455	Deletion	OXv9ch01	3,061,774	3,061,886	112	KOv8ch01	3,479,732	3,479,732	0

Figure S9 Detected SVs between the genomes of Korso and the OX-heart. **a**, Size distribution of detected SVs. **b**, Ratios of transposable element contents in SV regions to those in the entire genomes. **c**, Integrative Genomics Viewer (IGV) screenshots showing an example of the detected SV supported by the PacBio reads. PacBio reads from Korso and OX-heart were aligned to OX-heart (left) and Korso (right) genome, respectively. The expected deletion can be seen on the alignments of Korso reads on OX-heart genome (top left), whereas the expected insertion appears on that of OX-heart reads on Korso genome (bottom right). We manually checked 250 randomly selected SVs and confirmed 245 (98%) of them under IGV.

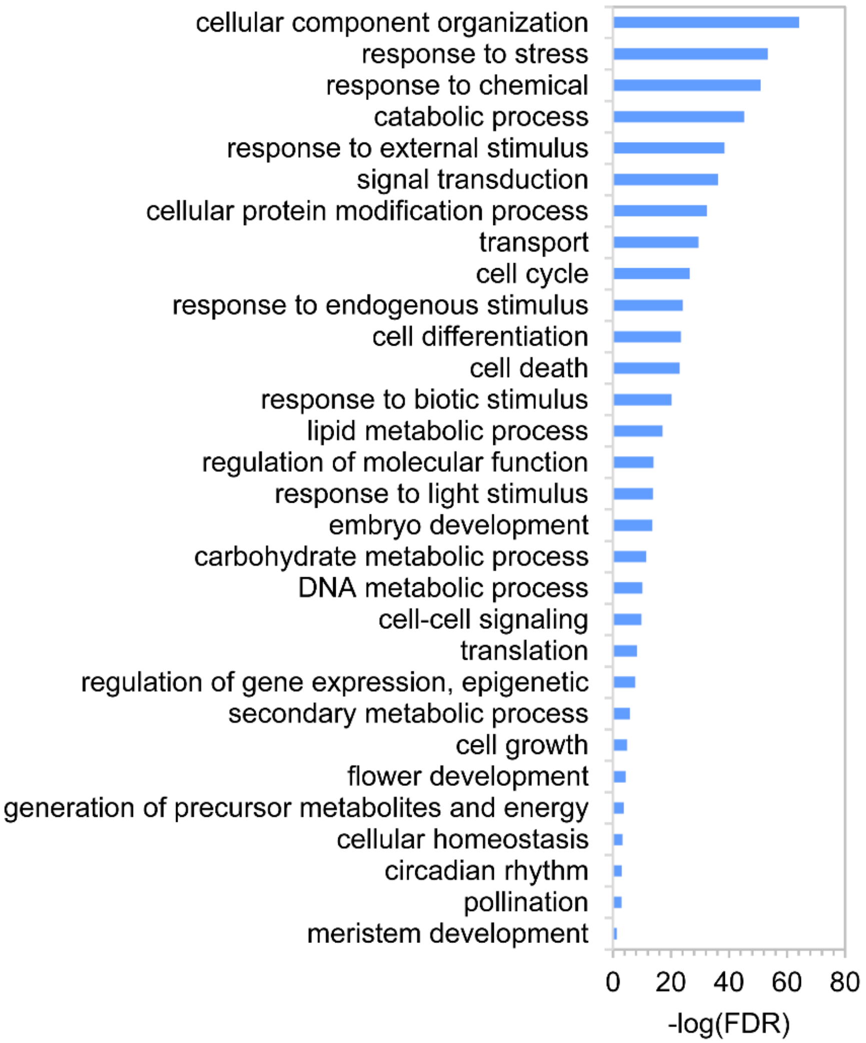

Figure S10 Significantly enriched GO terms in genes overlapping with detected SVs in their gene body and/or promoter regions

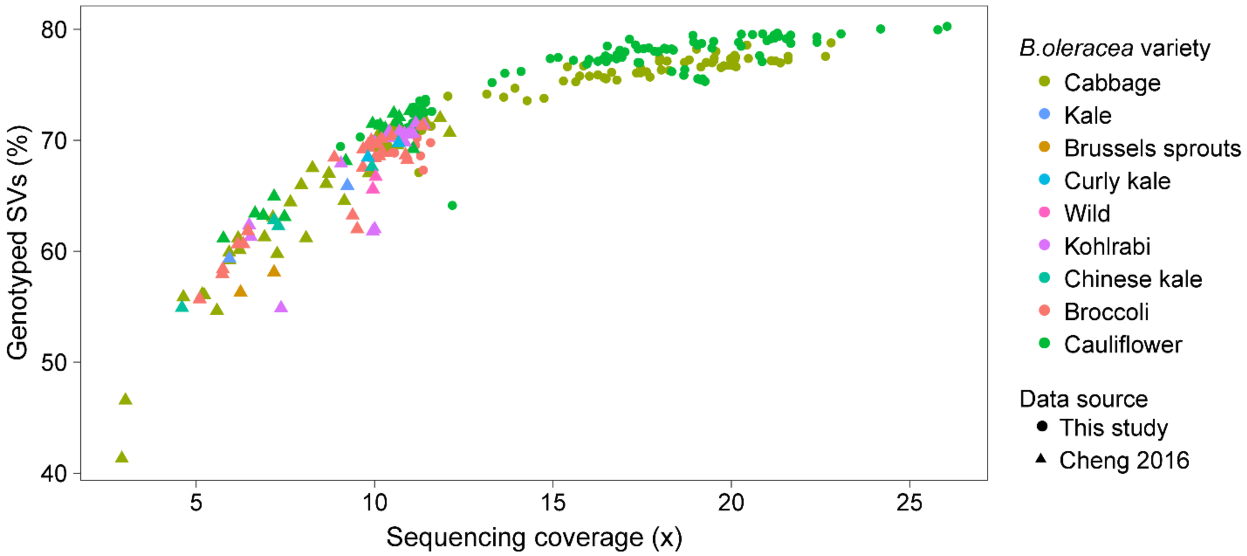

Figure S11 Distribution of SV genotyping rates in 271 *B. oleracea* accessions.

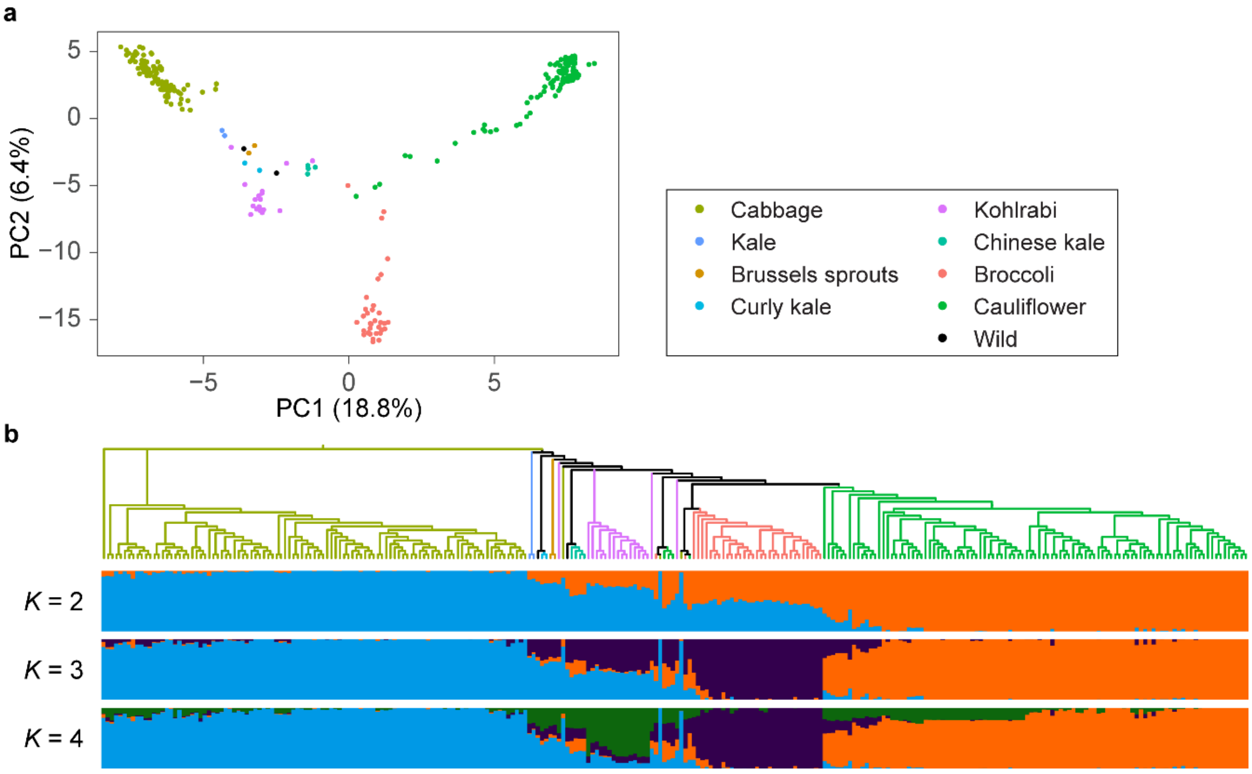

Figure S12 Population analysis of the 271 *B. oleracea* accessions using SNPs. **a,** Principal component analysis of *B. oleracea* accessions. **b**, Neighbor joining phylogenetic tree and population structure of the 271 *B. oleracea* accessions. Branch colors of the tree indicate different morphotypes as in **a**. *K*, number of ancestral kinships.

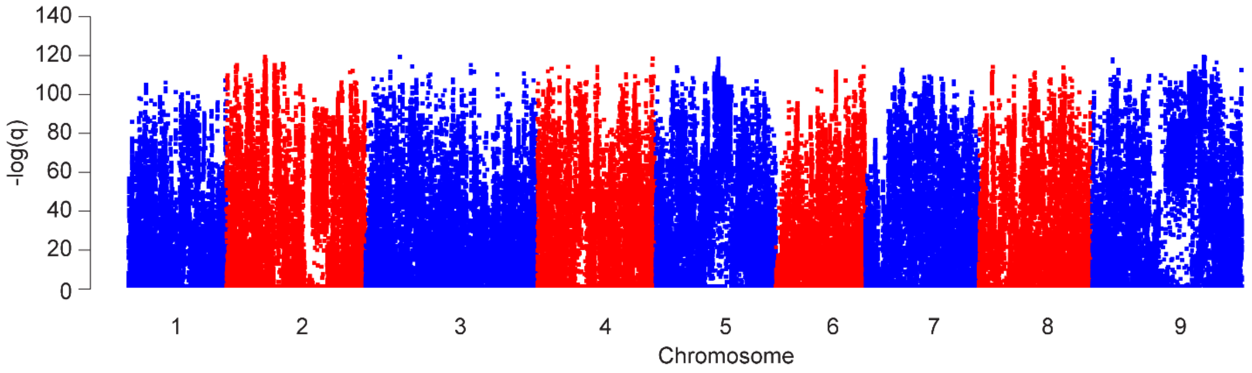

Figure S13 Manhattan plot displaying the -log(q-value) of allele variations between cabbage and cauliflower for SVs across the Korso genome. q-value, Bonferroni-corrected p value of Fisher's exact test.

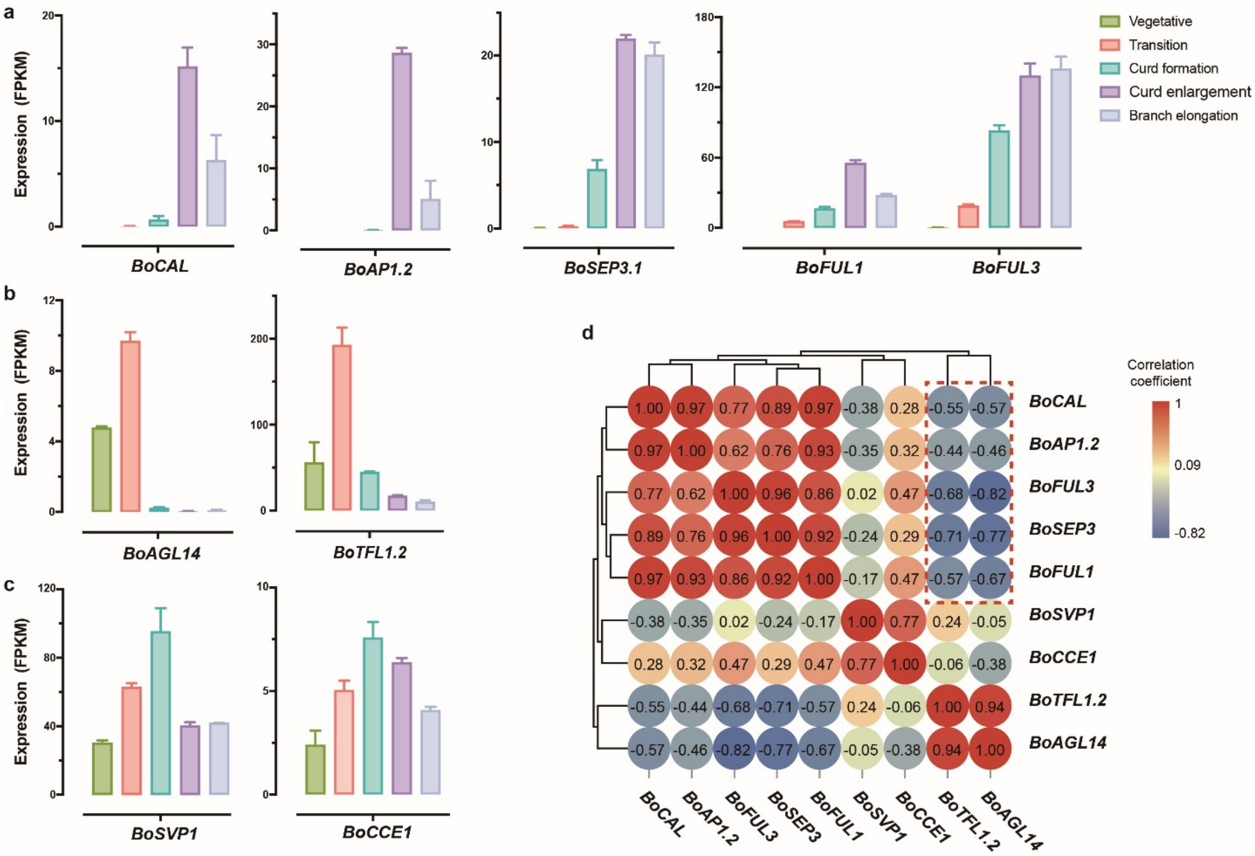

Figure S14 Expression profiles of genes potentially involved in curd maintenance and floral arrest. **a**, Expression profiles of FMI genes *BoCAL*, *BoAP1.2*, *BoSEP3.1*, *BoFUL1* and *BoFUL3*. **b**, Expression profiles of the IMI gene *BoTFL1.2* and its positive regulator *BoAGL14*. **c**, Expression profiles of the putative floral arrest genes *BoSVP1* and *BoCCE1*. **d**, Correlation heatmap of the gene expression profiles during curd development. The red dash square highlights the negative correlation between *BoAGL14*, *BoTFL1.2* and the FMI genes.
